# Nerves within bone and their application in tissue engineering of bone regeneration

**DOI:** 10.3389/fneur.2022.1085560

**Published:** 2023-02-02

**Authors:** Songyang Liu, Shen Liu, Shuming Li, Boran Liang, Xiao Han, Yonghui Liang, Xing Wei

**Affiliations:** Department of Orthopaedic, Aerospace Center Hosptial, Beijing, China

**Keywords:** nerves within bone, tissue engineering, bone regeneration, neuro-osteogenic network, peripheral nerves

## Abstract

Nerves within bone play an irreplaceable role in promoting bone regeneration. Crosstalk between the nerve system and bone has arisen to the attention of researchers in the field of basic medicine, clinical medicine, and biomaterials science. Successful bone regeneration relies on the appropriate participation of neural system components including nerve fibers, signaling molecules, and neural-related cells. Furthermore, more about the mechanisms through which nerves took part in bone regeneration and how these mechanisms could be integrated into tissue engineering scaffolds were under exploration. In the present review, we aimed to systematically elaborate on the structural and functional interrelationship between the nerve system and bone. In particular, peripheral nerves interact with the bone through innervated axons, multiple neurotrophins, and bone resident cells. Also, we aimed to summarize research that took advantage of the neuro-osteogenic network to design tissue engineering scaffolds for bone repair.

## 1. Introduction

During the process of bone development and regeneration, the non-osseous system including the nervous system, vascular system, and immune system plays an integral role. In particular, various types of nerves innervate into the bone and promote bone tissue regeneration by secreting a variety of molecules and interacting with bone lineage cells [e.g., osteoblasts and bone marrow stromal cells (BMSC)] and other cells that are colonized in the bone microenvironment (e.g., osteoclasts and vascular endothelial cells). The neural-bone network is also closely related to many bone-related diseases such as osteoporosis (1) and osteoarthritis (2). Despite the interdependent connection, most studies in the area of bone tissue engineering ignore the effect of the neural system.

**Figure 1:**
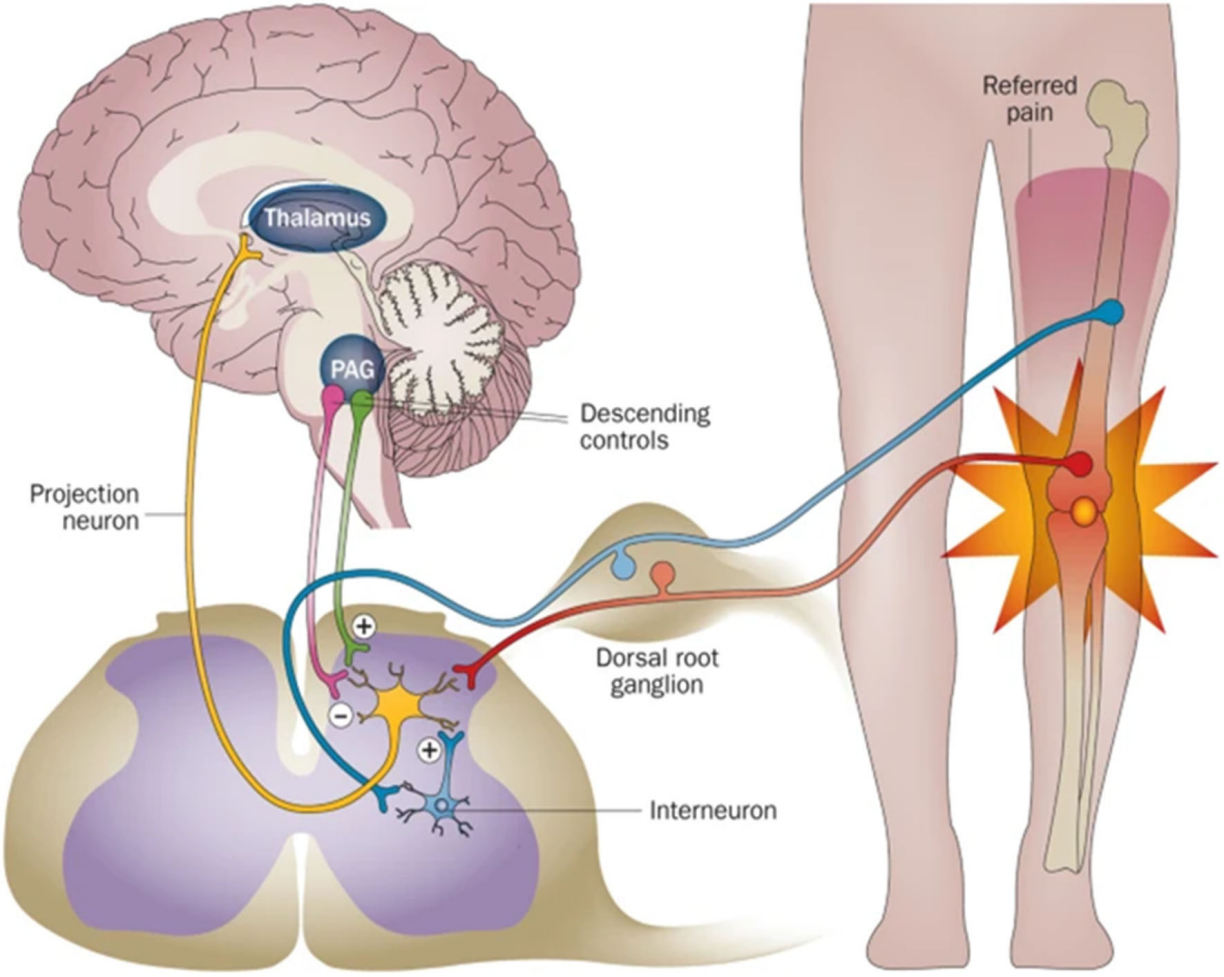
The scheme of the relationship between the nerve system and bone. PNS acts as a relay station to transmit information between CNS and the bone. Reprinted from Thakur et al. (7) with permission from Springer Nature.

Both the central nervous system (CNS) and peripheral nerve system (PNS) could act on bone metabolism through specific pathways. The CNS was first reported to regulate bone metabolism through leptin and its receptors (3). Also, CNS could affect bone mass through Neuropeptide Y (NPY), which exerts its effect *via* the endocrine pathway and sympathetic nervous output (4). Structurally, CNS neurons project nerve fibers to the spinal cord and DRG, where they assemble to form peripheral nerve fibers, which are innervated into bone (Figure 1). Peripheral nerves within bone are mostly divided into two types: sensory nerves and sympathetic nerves. The nerves are distributed in the periosteum, bone marrow, and cortical and trabecular bone (5, 6) and transform external information to CNS (Figure 1). As the nerve fibers of PNS were spatially connected with the bone, it is more instructive to consider PNS innervation in material design.

Although some reviews have summarized the crosstalk and interaction between bone and the nervous system, there are few researchers focused on reviewing the studies that applicated nerve-related elements to the field of bone tissue engineering. In the present review, we focus on summarizing recent research that explores the crosstalk between nerves and bone in the level of nerve fibers, molecules, and cells. We also review the research that applied the crosstalk between nerves and bone to design and fabricate bioactive bone scaffolds.

## 2. Distribution and function of nerves within bone

### 2.1. Distribution of the nerves within bone

**Figure 2:**
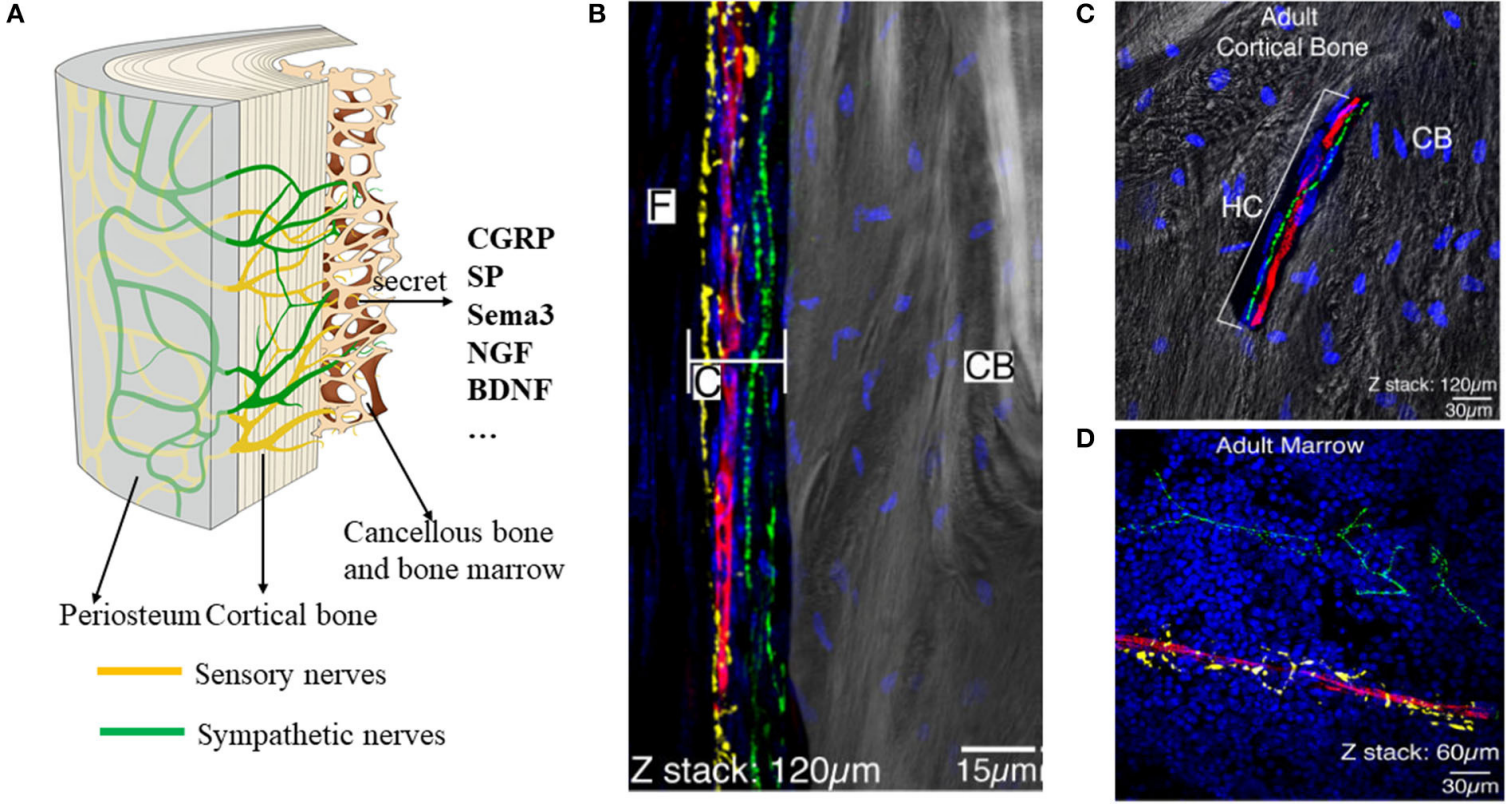
Distribution of the nerves within the periosteum, bone, and bone marrow. **(A)** Scheme of the nerves within the bone. There are mainly two types of nerves: sensory nerves and sympathetic nerves. **(B–D)** Representative images of the nerve fibers distributed in the periosteum **(B)**, cortical bone **(C)**, and bone marrow **(D)**. By immunofluorescence staining, TH+ sympathetic fibers showed yellow color, CGRP + fibers showed green, and CD31+ endothelial cells of blood vessels showed red. **(B–D)** Reprinted from Chartier et al. (8).

Neurons of the peripheral nervous system (PNS) converge to form ganglia, which radiates nerve fibers to innervate the bone (Figure 2A). There are only two types of nerve fibers within the bone: sensory nerves and sympathetic nerves (8, 9). The nerve fibers first infiltrate the periosteum where the fiber density is the greatest (Figure 2B). Then, nerves within the periosteum innervated into mineralized bone (cortical bone, cancellous bone) and bone marrow (Figure 2C) (8). In mineralized bone, nerve fibers were distributed along the Volkmann's and Haversian canals of bone (10, 11). Some fibers terminate blindly in the bone matrix, while others are in direct and intimate contact with bone lineage cells (11). In marrow space, sensory nerves (CGRP+) showed linear shape with varicose-rich endings and sympathetic nerves (TH+) rotate around the vessels (Figure 2D). In general, the nerve density is gradually decreased from the periosteum and cortical bone to the cancellous bone (12).

**Figure 3:**
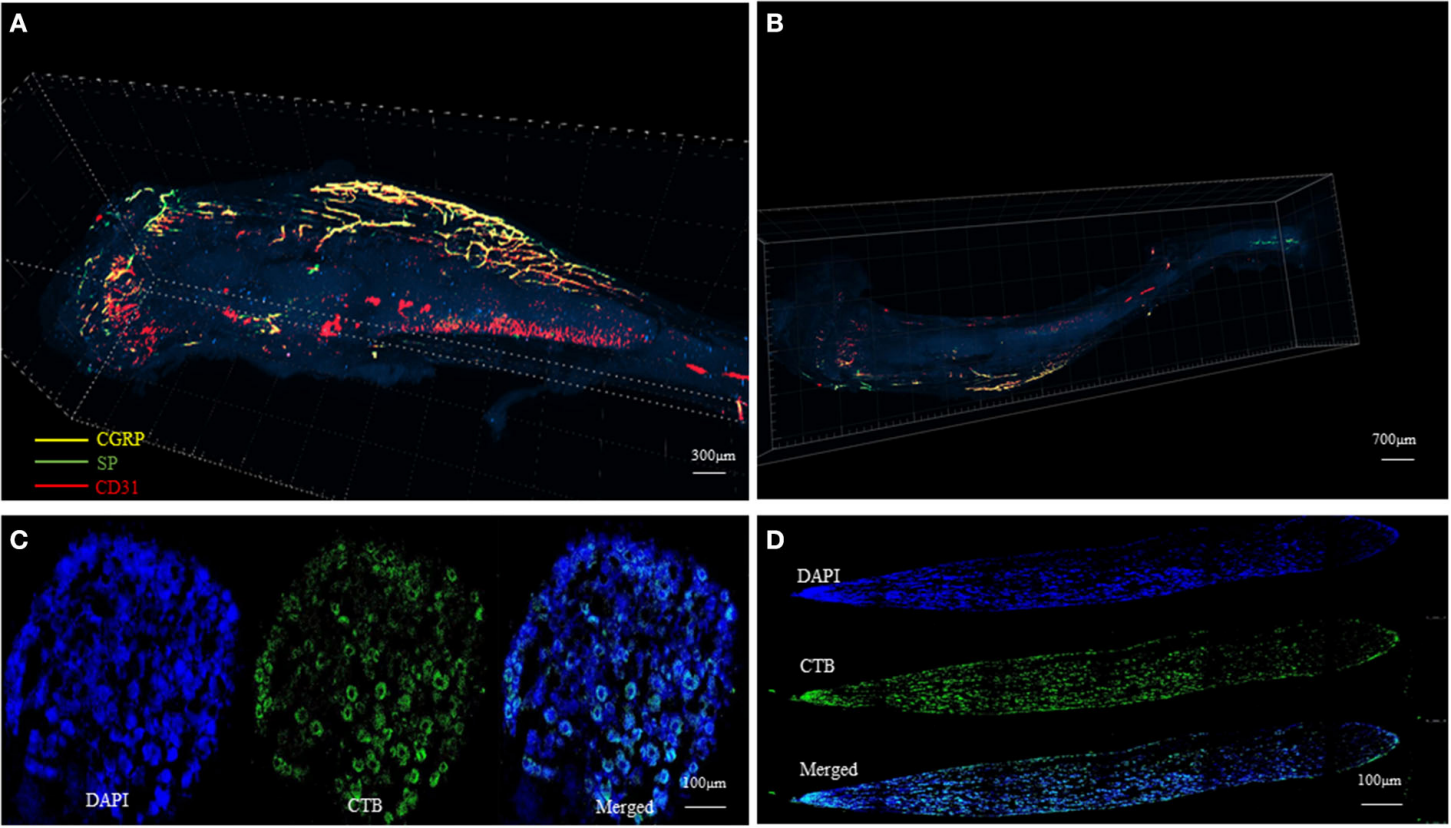
Tissue clearing of the mouse femur bone and intrabony nerves (Unpublished data). **(A, B)** Representative figures of intrabony nerves staining by tissue transparent staining. **(C, D)** Retrograde tracing of intramedullary nerves to the superior cervical neurons **(D)** and sympathetic ganglia **(C)**.

At present, studies on the distribution of nerve fibers mainly rely on two-dimensional immunological staining. We have developed a three-dimensional hard tissue clearing based on the PEGASOS method created by Jing et al. (14). The new method could provide a more straightforward view of the nerve distribution in the bone (Figure 3). More staining methods need to be developed to further study the structure and distribution of intrabony nerves and to provide ideas and theoretical support for the production of tissue engineering neuralized bone.

### 2.2. Function of the nerves within bone

As the intrabony nerves were mostly sensory and sympathetic, the main function of these fibers is related to perceiving pain (12, 15). They are closely related to pain caused by multiple diseases such as osteoarthritis, osteosarcoma, and Paget's disease (12). The type of pain depends on the injury mechanism and the bone site that was injured. For example, fracture induces stretching of the periosteum and cortical bone thus resulting in acute pain. Tumors or other lesions occupying the medullary cavity often caused chronic dull pain (16). When the noxious stimuli including acidosis and mechanical distortion happen, they first activate the intrabony nerves which are mainly A-delta fibers and C-fibers (15). These nerves processed the stimuli to the dorsal horn of the spinal cord and then to the brain where the sensation of pain occurs. There are other three mechanisms included in the occurrence of pain: peripheral sensitization, nerve sprouting, and central sensitization, which was detailed described in the review of “An understanding of bone pain: A narrative review” (15).

However, the research on the relationship between intrabony nerves and bone pain needs to be further deepened. On the one hand, the molecular mechanism and signaling pathway of connection between intrabony nerves and bone pain need to be further explored. On the other hand, the current strategies for repairing bone defects based on the theory of intrabony nerves focus more on the structural recovery of bone tissue while ignoring postoperative nerve recovery and pain relief.

In addition to sensing pain, nerves within bone also play an irreplaceable role in bone regeneration and repair after fracture. Aro et al. (13) found that the size and density of calli were smaller and less in the denervated fracture group compared with controls. Li et al. (17) reported that the number of CGRP-containing fibers increased significantly after fracture which might be beneficial for fracture healing and modeling. Fan et al. (18) implanted the sensory nerve tracts into TEP and demonstrated its effect on promoting osteogenesis in the rabbit femur defect model. Therefore, it is more prospective to consider the neurogenic osteogenesis of biomaterials in the future design of tissue-engineered bone scaffolds.

## 3. Signaling molecules from nerves to bone and their application in bone tissue engineering

The aforementioned intrabony nerves contact with various cells inside the bone including osteoblasts, osteoclasts, bone marrow stromal cells (BMSC), hematopoietic cells, endothelial cells of intramedullary blood vessels, and adipocytes (33–35). These nerve fibers could release a variety of signals that act locally on bone lineage cells and regulate bone metabolism (36). The signal molecules mainly contain neurotransmitters, neuropeptides, growth factors, and neuronal guidance factors, which work as bridges between the bone and nervous systems (Table 1).

**Table 1:** Molecules and application in tissue engineering.

**Neurotransmitter**	**Function**	**Downstream signal or receptor**	**Biomaterials**
CGRP	Stimulate osteoblast differentiation;	CAMP, FAK-VEGF	Mg (19, 20), PLGA (21)
Substance P	Pain transmission and analgesia	NK1	Chitin/PLGA (22), Titanium (23)
NE	Regulate bone mass	–	Collagen (24)
NPY	Regulate bone homeostatsis	Y1 receptor	–
**Growth factors**			
NGF	Regulating neuron survival and growth	TrkA, p-75NTR, RANKL	Calcium phosphate (25), Collagen (26)
BDNF	Promote bone fracture healing	TrkB	β-tricalcium phosphate (27),α-TCP + HA (28)
Sema3A	Affect osteoblast differentiation	mTOR	Hydroxyapatite scaffold (29, 30)
		Wnt	Silicon (31)
			PLGA (32)

### 3.1. Neurotransmitters

#### 3.1.1. Calcitonin gene-related peptide (CGRP)

Calcitonin gene-related peptide is a kind of representative neuropeptide released by sensory nerves within the bone (37). CGRP was originally discovered to be a potent vasodilator (38). Later, Oku et al. (39) found that it could produce a significant sensitization to pain. As for its effect on bone metabolism, He et al. (40) reported that CGRP could stimulate osteoblast differentiation and inhibit osteoclastogenesis. Sen et al. (41) demonstrated that CGRP administration could enhance new bone formation by recruiting BMSC to the ossification site.

In the field of tissue engineering, many researchers have shown that the role of CGRP cannot be ignored. Many studies have promoted bone formation by increasing local CGRP concentrations. Zhang et al. (19) found that implant-derived magnesium could promote osteogenic differentiation by increasing local CGRP levels, thus activating downstream cAMP. Li et al. (20) also demonstrated that magnesium could stimulate bone tissue repair through the CRGP–FAK–VEGF pathway. Combining CGRP directly with new biomaterials is another strategy, which was adopted by only a few studies. Yang et al. (21) constructed PLGA/Pda-EV scaffold which slowly delivered CGRP and demonstrate its effect on accelerating bone repair. For such studies, the sustained release and half-life of CGRP and specific mechanisms of interaction between CGRP and bone formation need to be further elucidated.

#### 3.1.2. Substance P

Substance P (SP) is a neuropeptide widely distributed in nerve fibers. When the nerve is stimulated, SP can be released at the central and peripheral nerve terminals and bind to NK1 receptors, which was expressed in bone lineage cells (42, 43). It is involved in the neurogenic inflammatory response and immune regulation and has the effects of pain transmission and analgesia (44, 45).

Amirthalingam et al. (46) combined Chitin/PLGA-CaSO_4_ with SP and demonstrated its promising function in promoting non-load-bearing bone defects by enhancing cell migration. SP has recently been found to recruit stem cells. Kim et al. constructed PLGA scaffolds encapsulated with SP and dexamethasone. The scaffolds were found to successfully treat calvarial bone defects by recruiting autologous stem cells and forming new bone tissues (22). Mu et al. (23) embedded SP on titanium substrates and found that they could facilitate bone healing through recruiting mesenchymal stem cells (MSCs). The limitation of the current studies is that the specific mechanism such as the signaling pathway by which SP-loaded scaffolds promote bone defect repair remains unclear.

#### 3.1.3. Norepinephrine

Norepinephrine (NE) is a neurotransmitter mainly synthesized and secreted by sympathetic postganglionic neurons and noradrenergic neurons in the brain. NE was reported to regulate bone mass through sympathetic signaling. For example, the knockout of the NE-synthetic enzyme could significantly increase bone mass (47).

Dhand et al. constructed bone-like scaffolds by electrospun collagen containing NE and Ca^2+^. The scaffolds showed excellent mechanical and biological properties for promoting bone regeneration (24).

#### 3.1.4. Neuropeptide Y

Neuropeptide Y (NPY) is a major neural transmitter that could be released by osteoblasts and peripheral nerves. It binds to the downstream Y1 receptor and regulates bone homeostasis (48). Park et al. (49) found reduced HSC numbers in the bone marrow of NPY-deficient mice, which might be explained by injury of sympathetic nerve fibers. Sousa et al. (50) demonstrated that deletion of the Y1 receptor negatively affects bone-resorbing activity.

The aforementioned studies all indicated the potential role of NPY in promoting bone regeneration, which is not yet properly valued and applicated in bone tissue engineering.

#### 3.1.5. Acetylcholine

Acetylcholine (Ach) is a kind of neurotransmitter released by cholinergic nerves. Once released, Ach mainly targeted osteoclasts through nicotinic acetylcholine receptors (nAchRs) on the cell surface (51, 52). *Mandl's* research found that agonists of nAchRs inhibited calcium oscillations in osteoclasts and blocked the RANKL-induced osteoclastogenesis, which lead to increased tibial bone volume in mice (53).

### 3.2. Growth factors and axon guidance molecules

#### 3.2.1. Nerve growth factor

Nerve growth factor (NGF) could promote the growth, development, differentiation, and maturation of central and peripheral neurons, maintain the normal function of the nervous system, and accelerate the repair of the nervous system after injury (54). In addition, elevated mRNA of NGF was found in injured bone (55). Research conducted by Mogi et al. (56) found that NGF worked as an apoptosis factor and promote the survival of osteoblastic cells. NGF could also induce osteoclastogenesis through a RANKL-independent pathway.

The nerve growth factor has two downstream receptors: the low-affinity receptor p75-NTR and high-affinity TrkA. Both receptors were first expressed on neurons to accompany the function of NGF in regulating neuron survival and growth. Later, they were found expressed on bone linage cells and involved in the bone repair process (57–59). Tomlinson et al. (60) found that the NGF-TrkA axis is of great importance to bone formation under mechanical loading in mice.

Taking advantage of its effects on repairing bone injury, some studies combined NGF with bone scaffolds. Jin et al. constructed a novel bone scaffold by combining porous biphasic calcium phosphate with NGF. *In vitro* experiments indicated its effects on enhancing osteoblast differentiation. *In vivo* experiments demonstrated that it could improve calvarial regeneration (25). Chen et al. incorporated collagen scaffold and applied it to repair the skull bone of mice. The results indicated that the scaffold could promote both neurogenesis and angiogenesis in bone defects (26).

There are limitations to applying NGF to the bone scaffold. On one hand, the high price limits its wide application. On the other hand, the half-life of NGF is short and it is hard to exert a long-term effect. In recent years, some studies have tried to replace NGF with its mimic peptide fragments (61), which mimic its activity and have a lasting effect.

#### 3.2.2. Brain-derived neurotrophic factor

Brain-derived neurotrophic factor (BDNF) is a neurotrophic protein first discovered in the pig brain in 1982 (62). Brain-derived neurotrophic factor and its receptors are widely expressed in the nervous system. Kilian et al. performed research to explore the role of BDNF in fracture healing. Their study showed that both BDNF and its receptor TrkB were over-expressed in human osteoblasts and endothelial cells, which is beneficial to bone fracture healing (63).

In addition to its application in nerve tissue engineering, BDNF was also widely used in constructing bone scaffolds in recent years. Liu et al. (27) found that when 100 ng/ml BDNF was combined with a β-tricalcium phosphate scaffold, the scaffold could promote hBMSC osteogenesis and neurogenesis *in vitro* and *in vivo*. Kauschke et al. incorporated BDNF in new bone cement and filled the fracture gaps with it. The results showed that the BDNF-functionalized composite could significantly promote fracture healing (28). However, the high price and short-term effect need to be solved in the future study.

#### 3.2.3. Semaphorin

Semaphorin (Sema3A) was a classic axonal guidance factor, which was reported to be highly expressed in both ends of the injured nerve after 3 weeks (64). As sensory nerves were innervated into the bone, Fukuda et al. (29) demonstrated that Sema 3A could regulate bone mass by affecting osteoblast differentiation.

In light of the effects of Sema 3A on bone remodeling, some studies constructed bone scaffolds with Sema 3A. Li et al. (29, 30) integrated Sema 3A and HIF1-α into a hydroxyapatite scaffold and test its effect on restricting bone defect. *In vitro* results showed the co-overexpression of Sema 3A and HIF1-α significantly increased the level of osteogenic and angiogenic genes. *In vivo* results also demonstrated that the complex scaffold boosted the new bone and collagen fiber formation of the mouse calvarial model. Ma et al. (31) found silicon-induced axon outgrowth and over-expression of Sema3A *in vitro*, which might be the mechanism through which sensory nerves promote bone defect healing. Liu et al. (32) engrafted Sema3A-modified ASCs into PLGA scaffolds and found that it could significantly promote bone formation through the Wnt pathway in a rat model.

## 4. The role of nerve-related cells in bone regeneration

### 4.1. Mesenchymal stem cells

Mesenchymal stem cells (MSCs) were a kind of stem cells derived from nerve crest and mesoderm, which have the ability to differentiate into a variety of cell types, including adipocytes, osteoblasts, and other cells. Due to their ability of multi-directional differentiation, it is widely used in the field of regenerative medicine (65). MSCs could be used by direct injection or combined with various tissue engineering scaffolds (66, 67). The application of MSCs in bone regeneration is described in detail in the reviews of Kangari (68) and Shang (69).

Recently, Carr identified a type of PDGFR^+^ mesenchymal precursor-like cells in injured nerve endoneurium which originate from the neural crest. Their research found that the neural crest-derived mesenchymal cells expand in number and differentiate into the bone during bone regeneration and repair (70, 71). It is worth noting that whether it can act in bone by directing MSCs to transform into neurons has not been investigated.

### 4.2. Schwann cells (SCs) and Schwann cell precursors (SCP)

Schwann cells play an essential role in the process of peripheral nerve regeneration and have been applied in nerve tissue engineering by many researchers (72–74). When Schwann cells were transplanted into the peripheral nerve injury site, it showed an excellent effect on the recovery of nerve function (72, 75). The potential role of assisting nerve regeneration rendered the possibility of using Schwann cells in bone defect repair. Cai et al. (76) performed a research and found that Schwann cells could enhance the proliferation and differentiation of osteoblasts. Although no studies transplanted SCs into bone defects, Schwann cell-derived exosomes were used in bone repair. Wu et al. found that Schwann cell-derived exosomes could significantly promote the migration, proliferation, and differentiation of BMSCs. The combination of Schwann cell exosomes with porous titanium implants can effectively improve its efficacy in repairing bone defects (77).

Schwann cell precursors (SCP) are a kind of neural-crest-derived cells and hold the potential for multidirectional differentiation. Xie et al. (78) reported that SCPs detaching from nerve fibers could differentiate into skeletal progenitors and mature osteocytes during embryonic development.

Since bone tissue itself does not contain Schwann cells and SCP, loading them into tissue engineering scaffolds to repair bone defects has not been studied. However, with the in-depth study of the interaction mechanism between Schwann cells and bone cells, relevant attempts can be made to combine SCs with bone scaffolds.

## 5. Conclusion and perspective

Nerves are widely distributed in the bone system, which is essential for perceiving pain and maintaining bone homeostasis. Nerve fibers, growth factors, and neural related cells are indispensable in the process of bone regeneration. Various bone scaffold has begun to take crosstalk between the nerve system and bone system into account. The idea of neurogenic bone regeneration has also been widely discussed. However, the mechanism of pain after bone regeneration is still under the cover. In addition, the interaction and specific signaling pathway between neural-related biomaterials and bone regeneration need to be further elucidated.

Many researchers have gradually recognized the role of neural-related factors in the construction of bone tissue engineering scaffolds and put them into practice, which was reviewed in the present article. Future studies should strengthen the cooperation of neuroscientists, tissue engineering experts, and bone researchers to come up with new strategies and propose new optimization strategies to repair bone defects and achieve better results.

## Author contributions

SoL, SheL, and XW wrote and edited the manuscript. ShuL, BL, and XH were also involved in drafting the manuscript and revising it critically for important intellectual content. All authors contributed to the article and approved the submitted version.
